# PRKDC mutations associated with immunodeficiency, granuloma and aire-dependent autoimmunity

**DOI:** 10.1186/1546-0096-12-S1-P42

**Published:** 2014-09-17

**Authors:** Alexandre Belot, Anne-Laure Mathieu, Estelle Veronese, Gillian Rice, Fanny Fouyssac, Yves Bertrand, Capucine Picard, Jolan Walter, Luigi Notarangelo, Catharina Schuetz, Heloise Reumaux, Mirjam Van Der Burg, Helen Kemp, Isabelle Rouvet, Christophe Malcus, Nicole Fabien, Yanick Crow, Christine Menetrier-Caux, Jean-Pierre De Villartay, Thierry Walzer

**Affiliations:** 1 Pediatric Nephrology, Rheumatology and Dermatology, Hospices Civils de Lyon, France; 2 U1111, INSERM, Lyon, France; 3 U1052, INSERM, Lyon, France; 4 Genetic Medicine, Manchester Academic Health Science Centre, University of Manchester, Manchester, UK; 5 Hémato-oncologie pédiatrique Hôpital d'Enfants , CHU Nancy, Nancy, France; 6 Institut d'hématologie et d'oncologie pédiatrique , Hospices Civils de Lyon, Lyon, France; 7 Study Center for Primary Immunodeficiencies, IMAGINE, Necker, Paris, France; 8 Division of Allergy/Immunology, Harvard Medical School, Boston, USA; 9 Division of Immunology, Boston Children's Hospital and Harvard Stem Cell Institute, Boston, USA; 10 Department of Pediatrics and Adolescent Medicine, University Medical Center Ulm, Ulm, Germany; 11 Pediatric Rheumatology Unit, Jeanne de Flandre Hospital, Lille, France; 12 Deptartment of Immunology, Erasmus MC, University Medical Center Rotterdam, Rotterdam, Netherlands; 13 Department of Human Metabolism , The Medical School University of Sheffield , Sheffield, UK; 14 Biotechnology, Hospices Civils de Lyon, Lyon, France; 15 Immunology Department, Hospices Civils de Lyon, Lyon, France; 16 Immunology, Hospices Civils de Lyon, Lyon, France; 17 U1163, INSERM, Paris, France

## Introduction

*PRKDC* encodes for DNA-dependent protein kinase catalytic subunit (DNA-PKcs), a kinase that forms part of a complex (DNA-PK) crucial for DNA double-strand break (DSB) repair and V(D)J recombination. In mice, DNA-PK also interacts with the transcription factor AIRE (autoimmune regulator) to promote central T cell tolerance.

## Objectives

We sought to understand the causes of an inflammatory disease with granuloma and autoimmunity, associated to decreasing T and B cell counts over time diagnosed in two unrelated patients.

## Methods

Genetic, molecular, and functional analyses were performed to characterize an inflammatory disease evocative of a combined immunodeficiency.

## Results

We identified *PRKDC* mutations in both patients. These patients exhibited a defect in DNA DSB repair and V(D)J recombination. Circulating T cells had a skewed cytokine response typical of Th1 and Th2 profiles. Moreover, mutated DNA-PKcs failed to promote AIRE-dependent transcription of peripheral tissue antigens *in vitro*. The latter defect correlated *in vivo*, with the production of anti-Calcium Sensing Receptor (anti-CaSR) autoantibodies, which are usually found in AIRE-deficient patients.

## Conclusion

Deficiency of DNA-PKcs, a key AIRE partner, can present as an inflammatory disease with organ-specific autoantibodies and these findings highlight the essential role of DNA-PKcs in regulating autoimmune responses and maintaining AIRE-dependent tolerance in human.

## Disclosure of interest

None declared.

